# Predictors of return to work with and without restrictions in public workers

**DOI:** 10.1371/journal.pone.0210392

**Published:** 2019-01-17

**Authors:** Adriano Dias, João Marcos Bernardes, Sandro Augusto Servilha Coquemala, Juan Gómez-Salgado, Carlos Ruiz-Frutos

**Affiliations:** 1 Public Health Graduate Program, Botucatu Medical School, Univ Estadual Paulista (UNESP), Sao Paulo, Brazil; 2 Technical Assistant, Botucatu Medical School, Univ Estadual Paulista (UNESP), Sao Paulo, Brazil; 3 Department of Nursing, University of Huelva, Huelva, Spain; 4 Safety and Health Posgrade Program, Universidad Espíritu Santo, Samborondón (Guayaquil), Ecuador; 5 Department of Preventive Medicine and Public Health, University of Huelva, Huelva, Spain; Ministry of Health and Sports, MYANMAR

## Abstract

**Background:**

Sick leaves are important events for both the worker and the employer. Many factors are related with sick leaves and depending on the factors the worker could perform a successful return to work. In this sense, the objective of this study is to identify those factors associated with return to work after sick leaves in a group of public workers in Brazil.

**Methods:**

A case-control study of return to work after sick leaves in a university campus from 2010 to 2015. Logistic regression models were adjusted for two different response variables: return to work with and without restrictions. A digital database was created and completed with data from manual sources.

**Results:**

A computerised database has been created, based on manual records, which has allowed us to identify labour and non-labour factors associated with the return to work after a sick leave and the possible functional readaptation, with or without restrictions, in public workers. Age at the beginning of the process, number of sick leaves, those of more than 16 days, average duration (total time of sick leaves / number of medical records), and mid-level healthcare positions were associated with return to work without restrictions. In the model of return to work with restrictions, the age of hiring by the university, the number of sick leaves, those of more than 16 days, and mid-level healthcare positions, both rural work and operational positions, were associated to the response variable.

**Conclusions:**

This study has allowed us to identify the factors associated with the return to work after a period of sick leave in a large group of public workers. However, more research is needed on the mental disorders that cause sick leaves, their evaluation and the handling of these situations.

## Introduction

Sick leaves are important events from both the point of view of the worker and that of the employer, generating economic, social, and health burden [1]. Among the immediate effects of absenteeism we find the decrease of the working capacity and the loss of productivity [2–4] and, depending on the severity of the illness or its chronicity, the worker can return to work (RTW) with or without restrictions. Long-term sickness absence is one of the main risk factors for definitive removal from the labour market[5]. Studies on absenteeism in the public health system have shown a higher frequency in female workers between 30 and 45 years old, alternating the predominant disease in accordance with the activities carried out and the professionals involved[3,4,6,7]. On the other hand, changes in the working environment are determinants of sick leaves in all workers [8] to the extent that, at the present time, it is difficult to differentiate the sick leaves simply because of the type of employment contract [9]. In Brazil, about four million and five hundred thousand social security benefits were granted in 2015, of which nearly two million were due to work-related diseases or accidents. These social security benefits could be temporary (for work accidents or diseases–occupational or not) and/or definitive (for disability retirement). In the state of São Paulo, between 2003 and 2006, almost two hundred thousand sick leaves were granted per year for statutory staff of all the institutions linked to the Secretaries of State [3].

Long-term sickness absence is usually composed by repeated periods of short-term sick leaves that could increase in duration, interspersing periods with and without work absences [5,10]. Depending on the sick leave’s cause, the worker can return to work by performing activities different from those performed before the sick leave, even at different locations. Previous studies indicate that some factors are related to a successful RTW: age [11,12], sex [8], marital status [12], educational level [12,13], economic conditions [12], severity of the disease [11,14,15], onset of the treatment [11], duration of the illness and/or the sick leave [12], chronicity of the disease/multiplicity of sick leaves [13–17], level of commitment [14,16], duration and type of work [15], and motivational interventions towards the RTW [13,14,17,18].

The objective of this study was to quantify the factors associated with RTW, with and without restrictions among public workers. In addition to the scarce literature on some of RTW determinants such as mental illnesses [19], this type of approach can facilitate decision-making by anticipating the course of its evolution, thus minimising its negative effects for both the worker and the employer.

## Material and methods

### Design, field of study, population, and sample

This is a case-control study on return to work after sick leaves among all statutory workers of a University Campus located in inland São Paulo state who were absent from work due to an illness between the months of January 2010 and December 2015. We retrospectively studied data on all workers who were on sick leave during the before mentioned period.

Workers were divided into three groups, two cases groups and one control group: a) cases type 1 (non-restricted workers): those who returned to work with different functions from the ones they performed before the sick leave; b) cases type 2 (restricted workers): those who returned to work in functions other than those performed before the sick leave, but with physical or functional limitations that prevented them from performing certain work activities; and c) controls: workers who returned to work in the same roles they had before the sick leave period.

An analysis of all medical reports from the institution’s occupational medical service that examines all statutory workers who request a sick leave with a duration of two or more days was done to allocate the workers into one of the three groups. This service, that manages all sick leaves, is the only one that can grant or deny them. In addition, it is responsible for the medical boards that decide on RTW cases and it is composed by general practitioners and psychiatrists.

It should be highlighted that, during the period of data collection (which was done by the University’s health technical section), the university campus was composed by four university units (agricultural sciences, human health, animal health and reproduction, and biological sciences), an administrative unit, and a university hospital.

### Data collection

For the development of the study’s database, information was extracted from two institutional databases: the Integrated Occupational Management Software (IOM), which identifies the socio-demographic characteristics and functional aspects of workers and stores physician's evaluation data, and the Healthcare Medical System (HMS), which records information about RTW cases and their characteristics. These databases are not integrated or built under the same computer architecture, neither programmed for research purposes. This forced us to evaluate each RTW case, registered on the HMS, on a one-by-one basis to match them with the physician's reports performed during the period, registered on the IOM.

### Variables

The socio-demographic variables were: place of birth (São Paulo State or outside), sex, age at recruitment by the University and current age, and marital status (showed by two different variables). The functional characteristics were: type of work (13 categories), work unit, time since employment in the institution, and total time since employment. The characteristics of the sick leaves were: duration, number of sick leaves taken in the period, total duration of sick leaves (and the total time stratified), reason in accordance with the tenth edition of the international statistical classification of diseases, injuries and causes of death (International Classification of Diseases / ICD-10), and behaviour of repeated sick leaves (throughout the ICD chapters in repeated events). The RTW was described in terms of whether it took place, time until it happened and whether its implied restrictions or not (leading to two response variables, RTW with restrictions and RTW without restrictions), mean time (total time divided by number of sick leaves spells), and duration of the first spell. Also, the reasons for each sick leave spell were studied and, being multiple, they were defined as the first cause or the main one pointed out by the physician.

To this end, it was necessary to create data mining tools to discover the reasons for the sick leave in a qualitative verbatim base, in the same way as the variables related in the expert reports and those also described in the characteristics of sick leaves spells: type of injury, affected part, and presence of psychological symptoms.

### Statistical analysis

The exploratory analysis of the data was carried out by means of simple and accumulated percentage distributions, measures of central tendency and dispersion (for discrete and continuous variables, respectively). The comparisons between the groups were performed through chi-square and Z-tests when the variables were discrete, and through ANOVA, followed by Tukey's post-hoc tests, for continuous variables.

Predictive variables of RTW with and without restrictions were analysed in two steps by using multiple logistic regression models. In the first step, univariate logistic regression models were run for each of the potentially predictive variables. Variables that produced p-values ≤ 0.25 in the first step were entered the multiple models. These multiple logistic regression models were adjusted with stepwise insert criteria and p < 0.05 maintenance for the final adjustment [20]. All analyses were performed with the IBM/SPSS Statistics software, v. 20.0.

### Ethical aspects and data availability

The Helsinki declaration has been taken into consideration.

The Department of Occupational Health, Safety of Workers and Environmental Sustainability (COSTSA) authorised the only manipulation of the data by the responsible investigator, through protocol 1.874.625 (of 19^th^ December, 2016), after the evaluation and permission of the Ethics Committee of the Botucatu Medical School, Sao Paulo, Brazil.

Each of the Health Technical Units involved in this study gave their authorisation for the use of the data, also required by the Ethics Committee, following the original regulations and guidelines of the COSTSA. The authorisation for the use of the worker's data is restricted to be manipulated only by the person responsible for the study and, if this rule is broken, they will not consent to do any analysis and subsequent publication.

These data are confidential and protected by legal restrictions for having medical data of workers. Once the data was received, the subjects were anonymised and their names were taken out from the database before sending the information to the researcher. As the data was received anonymised, the Ethics Committee released the research team from the application of the consent term.

The COSTSA is a body governed directly by the Rectorate of the Paulista State University/UNESP, who has not direct relationship with the Research Team that performed the epidemiological study. Therefore, neither the researcher nor the local Ethics Committee have intellectual property over the data, and COSTSA reserves the right to maintain the confidentiality thereof, except for the results of the analysis presented in the article, following the legislation itself and the institutional regulations. The responsible researcher does not have the authority to request authorisation to send the database to any third party.

In this sense, the data cannot be deposited in a public repository or assigned for public use. To support this communication, Resolution No. 196/96 of the National Health Council of Brazil for the approval of directives and research standards that involve human beings establishes in article IV.1. e) the importance of the main investigator, guaranteeing the secrecy and privacy of the participants during all research phases; and, in this case, the Ethics Committee holds the duty to maintain the confidentiality of all the data obtained in the execution of the task and to file the complete protocol.

On the other hand, regarding authors and data use consent, the COSTSA director signs, with the approval of the Research Ethics Committee, that these data are confidential and cannot be made available to the parties or repositories.

## Results

A total of 965 workers and 5776 sick leaves of two days or more were included. There were 56 (5.8%), 195 (20.2%), and 714 (74%) workers without restrictions (WR), with restrictions (R) and control (C) groups, respectively.

Table 1, which presents the sociodemographic characteristics, shows that the groups were different in terms of: marital status, living with a partner, positions held, and age at hiring. This is proven by the fact that the R group was older when started working at the university, and the C and WR groups kept a higher proportion of fixed partner, as well as roles and positions of higher educational levels. Women represented similar percentages among the three groups: 61.6% of C (440), 62.5% of WR (35), and 66.7% of R (130), and there were no statistically significant differences between the three types of groups in relation to origin, unit, and time at work.

**Table 1:** Sociodemographic and working characteristics of the study population distributed in three types according to sick leaves RTW between 2010 and 2015.

		C		WR		R		
		n (714)	%	n (56)	%	n (195)	%	p-value*
Sex	Female	440_a+_	61.6	35_a_	62.5	130_a_	66.7	
	Male	274_a_	38.4	21_a_	37.5	65_a_	33.3	0.435
Marital status	Married	405_a_	56.7	22_b_	39.3	108_a_	55.4	
	Separated	80_a_	11.2	11_a,b_	19.6	38_b_	19.5	
	Divorced	40_a_	5.6	6_a_	10.7	11_a_	5.6	
	Single	179_a_	25.1	17_a_	30.4	36_a_	18.5	
	Widowed	4_a_	0.6	0_a_	0.0	2_a_	1.0	
	Others	6_a_	0.8	0_a_	0.0	0_a_	0.0	0.017
Has a partner	No	306_a_	42.9	34_b_	60.7	87_a_	44.6	
	Yes	408_a_	57.1	22_b_	39.3	108_a_	55.4	0.035
Origin	São Paulo	703_a_	98.5	55_a_	98.2	194_a_	99.5	
	Another state	11_a_	1.5	1_a_	1.8	1_a_	0.5	0.521
University units	Administration	33_a_	4.6	1_a_	1.8	7_a_	3.6	
	Agricultural sciences	49_a_	6.9	2_a_	3.6	10_a_	5.1	
	Human health	535_a_	74.9	51_b_	91.1	162_b_	83.1	
	Animal health	44_a_	6.2	1_a_	1.8	11_a_	5.6	
	Biological sciences	53_a_	7.4	1_a,b_	1.8	5_b_	2.6	0.062
Type of work	Administration	127_a_	17.8	4_b_	7.1	12_b_	6.2	
	Rural work	32_a_	4.5	1_a_	1.8	12_a_	6.2	
	Teaching	53_a_	7.4	0_b_	0.0	1_b_	0.5	
	Mid-level healthcare	224_a_	31.4	34_b_	60.7	116_b_	59.5	
	Operational	78_a_	10.9	7_a,b_	12.5	34_b_	17.4	
	Others mid-level	10_a_	1.4	3_b_	5.4	3_a,b_	1.5	
	Others high-level	21_a_	2.9	0_a_	0.0	1_a_	0.5	
	Radiotherapy	11_a_	1.5	1_a_	1.8	1_a_	0.5	
	Healthcare	56_a_	7.8	1_a,b_	1.8	4_b_	2.1	
	Supervisor	14_a_	2.0	0_a_	0.0	1_a_	0.5	
	Academic support	62_a_	8.7	5_a_	8.9	5_b_	2.6	
	Transport	8_a_	1.1	0_a_	0.0	1_a_	0.5	
	Supervision and reception	18_a_	2.5	0_a_	0.0	4_a_	2.1	<0.001
		**Mean**	**SD**	**Mean**	**SD**	**Mean**	**SD**	p-value**
Age of hiring by the university		28.42_a_	6.68	27.18_a_	6.55	29.93_b_	6.87	0.005
Time working at university		21.39_a_	8.06	20.29_a_	7.36	20.38_a_	6.01	0.184

*chi-square

**ANOVA

^**+**^Subscript letters (a,b,c): different letters show statistically significant differences between groups (by Z or Tukey test).

^+^ C: control; WR: RTW without restrictions; R: RTW with restrictions

Table 2 shows the differences between the groups regarding sick leaves, especially in terms of age at the onset of the process, total duration of the sick leave, duration of leave per episode, or number of medical evaluations performed during sick leaves of 16 days or more.

**Table 2:** Characteristics of sick leaves by illness distributed in three types according to RTW between 2010 and 2015.

		C		WR		R		
		n	%	n	%	n	%	p-value^1^
Number of medical evaluations in the period (n = 965)	1	203_a+_	28.4	2_b_	3.6	22_b_	11.3	
	2	148_a_	20.7	3_b_	5.4	11_b_	5.6	
	3	93_a_	13.0	4_a,b_	7.1	14_b_	7.2	
	4	54_a_	7.6	3_a_	5.4	16_a_	8.2	
	5	51_a_	7.2	2_a,b_	3.6	5_b_	2.6	
	6–10	96_a_	13.4	11_a_	19.5	33_a_	16.9	
	11–20	44_a_	6.2	15_b_	26.8	53_b_	27.2	
	21–59	25_a_	3.5	16_b_	28.6	41_b_	21.0	<0.001
ICD-10 chapter (n = 5776)	1	95_a_	3.1	4_b_	0.6	31_b_	1.5	
	2	166_a_	5.4	3_b_	0.5	50_c_	2.5	
	3	12_a_	0.4	0_a.b_	0.0	1_b_	0.0	
	4	59_a_	1.8	4_b_	0.6	20_b_	1.0	
	5	920_a_	29.8	351_b_	52.8	751_c_	37.2	
	6	87_a_	2.8	12_a.b_	1.8	32_b_	1.6	
	7	146_a_	4.7	13_b_	2.0	56_b_	2.8	
	8	14_a_	0.5	23_b_	3.5	11_a_	0.5	
	9	145_a_	4.7	21_a_	3.2	79_a_	3.9	
	10	79_a_	2.6	7_b_	1.1	96_c_	4.8	
	11	129_a_	4.2	16_b_	2.4	44_b_	2.2	
	12	50_a_	1.6	13_a_	2.0	33_a_	1.6	
	13	399_a_	12.8	117_b_	17.5	515_c_	25.5	
	14	104_a_	3.4	5_b_	0.8	59_a_	2.9	
	15	15_a_	0.5	5_a_	0.8	2_b_	0.1	
	16	1_a_	0.1	0_a_	0.0	0_a_	0.0	
	17	11_a.b_	0.4	4_b_	0.6	3_a_	0.2	
	18	54_a_	1.7	10_a_	1.5	25_a_	1.2	
	19	244_a_	7.8	18_b_	2.7	100_c_	4.9	
	20	15_a_	0.5	0_a_	0.0	10_a_	0.5	
	21	347_a_	11.2	37_b_	5.6	103_b_	5.1	<0.001
Injured body part (n = 1018)**	Trunk	94_a_	17.5	38_b_	32.8	96_b_	26.4	
	Feet	69_a_	12.8	13_a_	11.2	48_a_	13.2	
	Legs	49_a_	9.1	11_a_	9.5	39_a_	10.7	
	Eyes	101_a_	18.8	8_b_	6.9	42_b_	11.5	
	Hands	87_a_	16.2	15_a_	12.9	60_a_	16.5	
	Fingers	27_a_	5.0	6_a_	5.2	4_b_	1.1	
	Head	48_a_	8.9	12_a_	10.3	27_a_	7.4	
	Arms	63_a_	11.7	13_a_	11.2	48_a_	13.2	<0.001
Type of injury (n = 1385)**	Sharp-cutting	8_a_	1.1	0_a_	0.0	7_a_	1.3	
	Dislocation	7_a_	1.0	0_a_	0.0	1_a_	0.2	
	Fracture	81_a_	11.6	9_b_	5.9	51_a,b_	9.5	
	Sprain	29_a_	4.2	0_b_	0.0	6_b_	1.1	
	Lethargy	1_a_	0.2	0_a_	0.0	1_a_	0.2	
	Pain	521_a_	74.7	143_b_	93.5	448_c_	83.7	
	Contusion	37_a_	5.3	1_b_	0.6	17_a,b_	3.2	
	Burn	13_a_	1.9	0_a_	0.0	4_a_	0.8	<0.001
Psychological symptoms (n = 5776)**	No	2311_a_	74.7	368_b_	55.5	1365_c_	67.5	
	Yes	781_a_	25.3	295_b_	44.5	656_c_	32.5	<0.001
ICD chapter modified (n = 965)	No	247_a_	34.6	18_a_	32.1	67_a_	34.4	
	Yes	467_a_	65.4	38_a_	67.9	128_a_	65.6	0.933
Symptoms start with chapter V of the ICD-10 and is kept until 20^th^ expert record (n = 965)	No	383_a_	53.6	17_b_	30.4	78_b_	40.0	
	Yes	331_a_	46.4	39_b_	69.6	117_b_	60.0	<0.001
Symptoms start with other chapter and change to chapter V of the ICD-10 until 20^th^ expert record (n = 965)	No	536_a_	75.1	54_b_	96.4	178_b_	91.3	
	Yes	178_a_	24.9	2_b_	3.6	17_b_	8.7	<0.001
Sick leave ≥ 16 days (n = 965)	No	388_a_	54.3	5_b_	8.9	62_c_	31.8	
	Yes	326a	45.7	51b	91.1	133c	68.2	<0.001
		**C**		**WR**		**R**		
		**Mean**	**SD**	**Mean**	**SD**	**Mean**	**SD**	**p-value**^**2**^
Age at the start of the process (n = 965)		49.81_a_	7.88	47.47_a_	7.27	50.31_b_	6.88	0.049
Total sick leave time in days (n = 965)		193.18_a_	464.25	818.02_b_	695.34	756.32_b_	1040.63	<0.001
Time until RTW in days (n = 239)		446.17_a_	876.64	599.01_a_	1100.07	564.47_a_	1053.95	0.351
Duration of sick leave (n = 5776)		193.18_a_	463.94	818.02_b_	689.42	756.32_c_	1038.09	<0.001
Mean time (total time of sick leave / Number of medical records) (n = 965)		40.03_a_	115.47	66.08_a.b_	85.60	65.93_b_	129.29	0.011
Days of sick leave in first episode		41.98_a_	159.10	43.43_a_	135.65	77.39_a_	258.51	0.055

^1^ chi-square.

^2^ANOVA

^**+**^Subscript letters (a,b,c): different letters show statistically significant differences between groups (by Z or Tukey test).

C: control; WR: RTW without restrictions; R: RTW with restrictions. ICD-10: International Classification of Diseases 10th Edition

* I—Certain infectious and parasitic diseases; II- Tumours (Neoplasms); III- Diseases of the blood and blood-forming organs and certain disorders involving the immune mechanism; IV- Endocrine, nutritional and metabolic diseases; V- Mental and behavioural disorders; VI-Diseases of the nervous system; VII- Diseases of the eye, adnexa. VIII- Diseases of the ear and mastoid process; IX- Diseases of the circulatory system; X- Diseases of the respiratory system; XI- Diseases of the digestive system; XII- Diseases of the skin and subcutaneous tissue; XIII- Diseases of the musculoskeletal system and connective tissue. XIV- Diseases of the genitourinary system; XV- Pregnancy, childbirth and the puerperium; XVI—Certain conditions originating in the perinatal period; XVII- Congenital malformations, deformations and chromosomal abnormalities; XVIII- Symptoms, signs and abnormal clinical and laboratory findings, not elsewhere classified; XIX- Injury, poisoning and certain other consequences of external causes; XX- External causes of morbidity and mortality; XXI- Factors influencing health status and contact with health services.

** Referred to by the doctor-expert in the record of the consultation and extracted from the text.

There were no differences between the three groups studied with respect to the duration of the sick leave in the first episode, to the accumulated time until RTW, or other modifications based on the chapters on diseases throughout the process, except for Chapter V (mental disorders).

In the groups where individuals returned to work in different functions (WR and R), the accumulated duration and the duration per sick leave episode were longer, and workers were older at the onset of the process, especially in the group that returned to work with limitations. In addition, WR and R presented higher proportions of long-term sick leaves and higher recurrence rates.

The groups were also statistically different in terms of the main disease chapter and in relation to mental disorders, either by the continuity of cases classified in chapter V of the ICD-10 or by migrating throughout the process towards that chapter, considering the interval between the 2^nd^ and the 20^th^ episode.

Regarding the information presented at the medical examination records where there was a reference to the body parts involved, the type of injury, and the presence of psychological symptoms, the groups also differed, since there was a greater proportion of references to these factors among those who had returned to work in different functions at the end of the process.

Table 3 presents the results of the simple logistic regression models between RTW without restrictions and each of the predictors. Age at hiring, age at the onset of the process, time since employment at the university, being a female, having a partner, number of medical evaluations, sick leave for 16 days or more, duration of sick leave in the first episode, total and average duration of sick leave, some work positions, and initiating the process of sick leave with diseases included in the mental disorders or in the ear and the mastoid process chapters of the ICD reached p-values ≤ 0.25 and, thus, were entered in the multiple model.

**Table 3:** Adjustment of simple logistic regression models. Odds Ratio, confidence intervals, and p-values, having as a result RTW without restrictions of university workers, between 2010 and 2015.

	OR	CI 95%		P-value
		LL	UL	
Age of hiring by the university	0.912	0.903	0.921	<0.001
Age at the start of the process	0.949	0.944	0.954	<0.001
Time working at university	0.888	0.876	0.901	<0.001
Total sick leave time	0.999	0.998	0.999	<0.001
Female (ref. Male)	0.375	0.314	0.449	<0.001
Having a partner (ref. no partner)	0.054	0.035	0.083	<0.001
Born out of the state of São Paulo (ref. São Paulo)	1.162	0.147	9.167	0.887
Number of medical evaluations	1.119	1.088	1.151	<0.001
Sick leave ≥ 16 days (ref. ≤15 days)	12.140	4.789	30.775	<0.001
Mean time (total leave time / Number of medical records)	1.001	1.000	1.003	0.121
University units				
Administration	1.000	—-	—-	—-
Agricultural sciences	1.347	0.117	15.464	0.811
Human health	3.146	0.421	23.480	0.264
Animal health	0.750	0.045	12.436	0.841
Biological sciences	0.623	0.038	10.297	0.741
Positions				
Administration	1.000	—-	—-	—-
Rural work	0.031	0.004	0.229	0.001
Teaching	0.000	0.000	—-	0.997
Mid-level healthcare	0.152	0.106	0.218	<0.001
Operational	0.090	0.041	0.194	<0.001
Others mid-level	0.300	0.083	1.090	0.067
Others high-level	0.000	0.000	—-	0.998
Radiotherapy	0.091	0.012	0.704	0.022
Healthcare	0.018	0.002	0.129	<0.001
Supervisors	0.000	0.000	—-	0.998
Academic support	0.081	0.032	0.201	<0.001
Transport	0.000	0.000	—-	0.999
Supervision and reception	0.000	0.000	—-	0.998
ICD-10 chapter in first episode				
I	1.000	—-	—-	—-
II	0.541	0.033	8.932	0.668
III	0.000	0.000	—-	0.999
IV	2.750	0.159	47.516	0.487
V	5.383	0.702	41.302	0.105
VI	3.474	0.295	40.900	0.322
VII	1.015	0.089	11.612	0.990
VIII	13.200	1.002	173.881	0.050
IX	2.152	0.214	21.615	0.515
X	1.375	0.082	23.097	0.825
XI	0.600	0.036	9.918	0.721
XII	4.400	0.370	52.375	0.241
XIII	4.605	0.576	36.825	0.150
XIV	0.917	0.055	15.253	0.952
XV	0.000	0.000	—-	1.000
XVI	0.000	0.000	—-	1.000
XVII	0.000	0.000	—-	0.999
XVIII	0.000	0.000	—-	0.999
XIX	1.375	0.138	13.720	0.786
XX	0.000	0.000	—-	0.999
XXI	1.650	0.098	27.874	0.728
Days of sick leave in first episode	0.950	0.941	0.960	<0.001

I- Certain infectious and parasitic diseases; II- Tumours (Neoplasms); III- Diseases of the blood and blood-forming organs and certain disorders involving the immune mechanism; IV- Endocrine, nutritional and metabolic diseases; V- Mental and behavioural disorders; VI-Diseases of the nervous system; VII- Diseases of the eye, adnexa. VIII- Diseases of the ear and mastoid process; IX- Diseases of the circulatory system; X- Diseases of the respiratory system; XI- Diseases of the digestive system; XII- Diseases of the skin and subcutaneous tissue; XIII- Diseases of the musculoskeletal system and connective tissue. XIV- Diseases of the genitourinary system; XV- Pregnancy, childbirth and the puerperium; XVI—Certain conditions originating in the perinatal period; XVII- Congenital malformations, deformations and chromosomal abnormalities; XVIII- Symptoms, signs and abnormal clinical and laboratory findings, not elsewhere classified; XIX- Injury, poisoning and certain other consequences of external causes; XX- External causes of morbidity and mortality; XXI- Factors influencing health status and contact with health services.

OR: Odds ratio. ICD-10: International classification of diseases 10th Edition.

Observing the variables included in the multiple regression model (Table 4), the age at the onset of the process proved to be a protective factor and was statistically significant (OR: 0.94). However, the number of medical evaluations performed during the period (OR: 1.06), the sick leaves of 16 days or more (OR: 1.89), the average sick leave duration (OR: 6.89) and working in mid-level healthcare work activities (OR: 5.31) proved to be risk factors, being the last two strongly associated with RTW without restrictions.

**Table 4:** Adjustment of the multiple logistic regression model. Odds Ratio, respective confidence intervals, and p-values, having as a result RTW without restrictions of university workers between 2010 and 2015.

	OR	CI 95%	
		LL	UL
Age at the start of the process	0.936	0.898	0.976
Number of medical evaluations	1.058	1.010	1.107
Sick leaves ≥ 16 days (ref. ≤15 days)	1.897	1.235	2.915
Mean time (Total sick leave time / Number of expert records)	6.892	2.545	18.666
Mid-level healthcare position	5.311	1.670	16.887

Table 5 shows the results of the simple logistic regression models between RTW with restrictions and each of the predictors. As can be seen, age at hiring, age at the onset of the process, time since employment at the university, being a female, having a partner, coming from outside of the state of São Paulo, working in a mid-level health positions, number of medical examination reports, sick leaves of 16 days or more, duration of the sick leave in the first episode, average sick leave duration, and initiating the process of sick leave due to diseases of the musculoskeletal system and connective tissue or due to a mental disease reached p-values ≤ 0.25 and, thus, were included in the multiple model.

**Table 5:** Adjustment of simple logistic regression model. Odds Ratio, confidence intervals, and p-values, having as a result RTW with restrictions of university workers, between 2010 and 2015.

	OR	CI 95%		P-value
		LL	UL	
Age of hiring by the university	0.959	0.954	0.964	<0.001
Age at the start of the process	0.975	0.972	0.978	<0.001
Time working at university	0.944	0.937	0.951	<0.001
Total sick leave time	1.000	1.000	1.000	0.667
Female (ref. Male)	0.295	0.243	0.359	<0.001
Having a partner (ref. no partner)	0.265	0.214	0.327	<0.001
Out of state of São Paulo (ref. São Paulo)	0.091	0.012	0.704	0.002
Number of expert records	1.120	1.095	1.145	<0.001
Sick leaves ≥ 16 days (ref. ≤15 days)	2.561	1.831	3.582	<0.001
Mean time (Total sick leave time / Number of medical records)	1.001	1.000	1.003	0.013
University units				
Administration	1	—-	—-	—-
Agricultural sciences	0.982	0.339	2.843	0.973
Human health	1.428	0.620	3.288	0.403
Animal health	1.179	0.413	3.367	0.759
Biological sciences	0.445	0.130	1.517	0.196
Position				
Administration	1	—-	—-	—-
Rural work	0.402	0.116	1.387	0.149
Teaching	1.594	0.445	5.706	0.474
Mid-level healthcare	0.080	0.008	0.767	0.029
Operational	2.201	0.724	6.692	0.164
Others mid-level	1.853	0.580	5.917	0.298
Others high-level	1.275	0.236	6.899	0.778
Radiotherapy	0.202	0.021	1.984	0.170
Healthcare	0.386	0.038	3.927	0.422
Supervision	0.304	0.069	1.345	0.116
Academic support	0.304	0.030	3.036	0.310
Transport of people and materials	0.343	0.083	1.418	0.139
Supervision and reception	0.531	0.051	5.553	0.597
ICD-10 chapter in first episode				
I	1	—-	—-	—-
II	0.757	0.223	2.574	0.656
III	0.000	0.000	—-	0.999
IV	1.650	0.341	7.983	0.534
V	2.876	1.071	7.720	0.036
VI	1.042	0.224	4.854	0.958
VII	1.422	0.471	4.286	0.532
VIII	1.320	0.127	13.758	0.816
IX	1.722	0.553	5.357	0.348
X	1.925	0.545	6.802	0.309
XI	1.080	0.333	3.498	0.898
XII	1.760	0.413	7.500	0.445
XIII	3.607	1.320	9.859	0.012
XIV	1.100	0.307	3.946	0.884
XV	3.300	0.251	43.470	0.364
XVI	0.000	0.000	—-	1.000
XVII	0.000	0.000	—-	0.999
XVIII	2.200	0.440	11.006	0.337
XIX	0.917	0.290	2.895	0.882
XX	1.100	0.108	11.155	0.936
XXI	0.330	0.036	3.031	0.327
Days of sick leave in first episode	1.001	1.000	1.002	0.024

The variables that predicted RTW with restrictions, according to the multiple regression model (Table 6), were: age at hiring by the university (OR: 1.03), number of medical evaluations performed (OR: 1.10), sick leaves of 16 days or more (OR: 1.56), working in rural activities (OR: 4.71), working in mid-level healthcare positions (OR: 5.62), and in operational positions (OR: 4.07). We highlight the strong association between occupying some work positions and the outcome in question, since it increased the risk of returning to work with restrictions between four and almost six times.

**Table 6:** Adjustment of the multiple logistic regression model. Odds Ratio, respective confidence intervals and p-values, having as a result RTW with restrictions of university workers, between 2010 and 2015.

	OR	CI 95%	
		LL	UL
Age of hiring by the university	1.033	1.001	1.068
Number of expert records	1.103	1.075	1.131
Sick leaves ≥ 16 days (ref. ≤15 days)	1.561	1.001	2.440
Type of work (Ref. Administration)			
Rural work	4.709	1.678	13.214
Mid-level healthcare	5.624	2.670	11.846
Operational	4.073	1.783	9.302

## Discussion

This study has allowed to investigate the factors associated with RTW after a period of sick leave among statutory workers of a public university in the state of São Paulo, Brazil. In the literature and in our study, we find reasonable discrepancies in the role of: the type of disease that causes the sick leave, job characteristics, management of the sick leave, personal characteristics of the worker, some extra labour factors, among others [21–25].

In our study, we have found a high percentage (37%) of sick leaves due to diseases within the mental disorders group, like that of other groups of Brazilian public workers [26,27]. However, this percentage is much higher than in other groups and working environments [22] and quite similar to others [28]. One hypothesis to explain this high percentage is the fact that psychiatrists performed some of the medical evaluations, what could induce a greater number of this type of diagnoses compared with other studies in which such specialists did not carry out the medical evaluations. In relation to RTW, the diseases within the mental disorders group represented 29.8% of the cases in which individuals returned to work in the same roles, 52.8% of the ones that individuals returned to work in different functions, and 37.2% of those who returned to work in different functions with restrictions. To our knowledge, this is the first study to assess the prevalence of RTW according to the disease that has caused the sick leave. Thus, we have no data with which to compare our study outcomes.

It is known, however, that the time to RTW after a sick leave due to mental disorders is long [29,30], being not only greater than regarding neoplastic conditions [31] and having one of the lowest rates of RTW. For example, in Denmark, 12.7% of these cases never return to perform their activities [32]. Diseases of the musculoskeletal system and of the connective tissue were the second most frequent group of diseases that caused sick leaves. This is worrisome since these two groups of diseases usually become chronic and associated [33,34], consequently increasing the periods of sick leave [34,35].

The age at the beginning of the process, which in our study appears as a protective factor in the cases of RTW in different functions, is something that coincides with previous studies on the predictors of a successful RTW [8,9], although it is not kept in the multiple regression model of RTW with restrictions. The age at hiring by the university is one of the variables that was identified as a risk factor in the multiple model of RTW with restrictions (OR = 1.03), but not in the multiple model of RTW without restrictions.

Even though the literature shows that being a female is associated with a successful RTW [8], in our study this variable was not kept in any of the final models.

We have seen differences between the three groups studied in relation to variables that, in the literature, have been related to a successful return to work, such as the marital status [12], although they were not kept in the final multiple regression models. At the educational level [12,13], we only saw that being a mid-level healthcare worker is one of the strongest risk factors in the final multiple regression models (more than 5-fold for both). However, it seems that this could be more related to the stressful and debilitating (physically and emotionally) professional activity carried out than to the educational level or even the economic conditions, another variable that was observed in previous studies [36].

The duration of the sick leaves is one of the variables that are kept as risk factors in both multiple regression models (1.9-fold for workers that returned to work without restrictions and 1.5 for those who returned with restrictions), as in other published studies [12].

In the medical evaluations reports of the individuals who returned to work in different functions (with or without restrictions), we see how there are more references to the injured body parts, the type of injury, and the presence of psychological symptoms. This thorough study on sick leaves seems logical given that, now, workers are not able to perform the same activities they developed in their original work place. However, no literature references have been found on this.

The relationship between the type of work or position held and the RTW is also described in the literature [15]. According to our results, working at rural, operational, and mid-level healthcare positions was strongly associated with the need to RTW with restrictions (more than 4-fold each). These situations could be explained by the greater difficulty that some types of work will present to workers after a continuous sick leave, injuries, or illnesses that reduce their work ability, because their activities could present a lot of specific and differentiated working conditions and technical or physical requirements, which are difficult to adapt without certain limitations with respect to the habitual activity. On the other hand, the position of mid-level healthcare worker is the only one that remains in the model of RTW without restrictions, probably due to the reasons explained before. It is important to remember that mid-level healthcare positions could assume a wide variety of activities and places in health services and in hospitals, helping RTW in different functions, possibly covering the limitations.

In the multiple regression model of RTW without restrictions, as well as in the model of the RTW with restrictions, the "amount of medical evaluations" appears as one of the significant risk factors (although weak), but we have not found this association in the literature as it is the type of sick leave that tends to require a greater number of evaluations until finding a job that is adapted to the capacities of the worker.

Among the limitations of the study, we find the use of data not only already registered (and, therefore, a lack of other information that could be useful), but also registered by different persons and with sources of information that required a manual work to complete the final database analysed. The use of sick leaves as a measure of morbidity has limitations because it tis multi-factorial and influenced not only by the health status of the individual, but also by the work environment, social and psychological factors, attitudes and commitment to work, as well as social insurance system. Secondly, the study population was derived from a single university, although from several different units, so whether the results of this research can be generalised to other groups of public servants is still unclear.

## Conclusions

The main conclusion of the study is related to its applicability, since its development has allowed building a computerised database from partially manual data sources, which has allowed identifying the factors associated with the RTW after a period of sick leave, with or without restrictions, in a large group of public workers. In this sense, several factors were identified, such as the age at the onset of the process (RTW without restrictions), age at hiring by the university (RTW with restrictions), amount of sick leaves, sick leaves of 16 days or more, having a mid-level healthcare post, to which two other types of positions must be added: "operational" or "rural" posts in cases of RTW with restrictions.

As future lines of research, we highlight to deepen in the causes of mental disorders and to further investigate the components of the three positions that have been identified as potential risk factors for returning to work in different functions, since early interventions may improve the prognosis and increase the probability of RTW in this cases [37]. In the same way, it will be necessary to study the influence of the sick leaves’ management [38] and the characteristics of the evaluating physicians during the process of RTW, as well as the level of commitment or the motivational interventions that could not be analysed in the present study. So, returning to work is a very complex situation that involves the relationship of many factors beyond the disease. Advanced knowledge on this issue can show whether these factors are common across all conditions and could form the basis for generic RTW strategies that can be tested and widely used within any condition and setting.

## Supporting information

S1 File Aggregated data. (PDF)
