# Supplementary material for: Predictors of return to work with and without restrictions in public workers
Source: PLoS One. 2019 Jan 17;14(1):e0210392. doi: 10.1371/journal.pone.0210392 (PMC6336309; doi:10.1371/journal.pone.0210392)
Supplement: S1 File — (PDF) [file pone.0210392.s001.pdf]

Predictors of return to work with and without restrictions in public workers

Descriptive results of the study variables

About sociodemographic and working characteristics

		n	%
Sex	Female	605	62.69
	Male	360	37.31
Has a partner	No	427	44.25%
	Yes	538	55.75%
Origin	São Paulo	952	98.65%
	Another state	13	1.35%
Unit	Administration	41	4.25%
	Agricultural sciences	61	6.32%
	Human health	748	77.51%
	Animal health	56	5.80%
	Biological sciences	59	6.11%
Type of work	Administration	143	14.82%
	Rural work	45	4.66%
	Teaching	54	5.60%
	Mid-level healthcare	374	38.76%
	Operational	119	12.33%
	Others mid-level	16	1.66%
	Others high-level	22	2.28%
	Radiotherapy	13	1.35%
	Healthcare	61	6.32%
	Supervisor	15	1.55%
	Academic support	72	7.46%
	Transport	9	0.93%
	Supervision and reception	22	2.28%
		**Mean**	**SD**
Age of hiring by the university		28.65	6.74
Time working at university		21.12	7.65

About characteristics of sick leaves by illness

		n	%
Number of medical evaluations in the period (n=965)	1	227	23.52%
	2	162	16.79%
	3	111	11.50%
	4	73	7.56%
	5	58	6.01%
	6-10	140	14.51%
	11-20	112	11.61%
	21-59	82	8.50%
ICD-10 chapter (n=5776)	1	130	2,25%
	2	219	3,79%
	3	13	0,23%
	4	83	1,44%
	5	2022	35,01%
	6	131	2,27%
	7	215	3,72%
	8	48	0,83%
	9	245	4,24%

	10	182	3,15%
	11	189	3,27%
	12	96	1,66%
	13	1031	17,85%
	14	168	2,91%
	15	22	0,38%
	16	1	0,02%
	17	18	0,31%
	18	89	1,54%
	19	362	6,27%
	20	25	0,43%
	21	487	8,43%
Injured body part (n=1018)	Trunk	228	22.40%
	Feet	130	12.77%
	Legs	99	9.72%
	Eyes	151	14.83%
	Hands	162	15.91%
	Fingers	37	3.63%
	Head	87	8.55%
	Arms	124	12.18%
Type of injury (n=1385)	Sharp-cutting	15	1.08%
	Dislocation	8	0.58%
	Fracture	141	10.18%
	Sprain	35	2.53%
	Lethargy	2	0.14%
	Pain	1112	80.29%
	Contusion	55	3.97%
	Burn	17	1.23%
Psychological symptoms (n=5776)	No	4044	70.01%
	Yes	1732	29.99%
ICD chapter modified (n=965)	No	332	34.40%
	Yes	633	65.60%
Symptoms start with chapter V of the ICD-10 and is kept until 20th expert record (n=965)	No	478	49.53%
	Yes	487	50.47%
Symptoms start with other chapter and change to chapter V of the ICD-10 until 20th expert record (n=965)	No	768	79.59%
	Yes	197	20.41%
Sick leave ≥ 16 days (n=965)	No	455	47.15%
	Yes	510	52.85%
Return to work (n=965)	without restrictions	56	5.81%
	with restrictions	195	20.21%
	control	714	73.98%
		**Mean**	**SD**
Age at the start of the process (n=965)		49.77	7.67
Total sick leave time in days (n=965)		343.24	684.99
Time until RTW in days (n=239)		523.89	896.64
Duration of sick leave (n=5776)		543.93	755.35
Mean time (total time of sick leave/ Number of medical records) (n=965)		46.78	117.36
Days of sick leave in first episode (n=965)		49.22	182.81
